# Three Calcium Hydroxylapatite‐Based Dermal Fillers Marketed in Mexico: Comparison of Particle Size and Shape Using Electron Microscopy

**DOI:** 10.1111/jocd.70100

**Published:** 2025-03-04

**Authors:** Gilberto A. Sanchez Rico, Silvia Beatriz Andrade Canto

**Affiliations:** ^1^ Oneline Beauty Clinic Cancún Quintana Roo Mexico; ^2^ Centro de Investigación Científica de Yucatán Mérida Yucatán Mexico

**Keywords:** biocompatibility, CaHa, calcium hydroxyapatite, dermal fillers, electron microscopy, particle shape, scanning electron microscopy, surface structure

## Abstract

**Background:**

Dermal fillers have been a popular choice for cosmetic dermatology procedures, and understanding the physical properties of microparticles used in dermal fillers is crucial for developing safe and effective aesthetic treatments.

**Aim:**

This study analyzed three dermal filler products (Radiesse, HarmonyCA, and Hydroxyfill) based on calcium hydroxyapatite microspheres approved for dermal filler procedures in Mexico.

**Material and Methods:**

The study used scanning electron microscopic analysis to study the morphology and characteristics of the calcium hydroxyapatite microspheres used in these dermal filler products.

**Results:**

Results revealed important differences in the shape of the microspheres between the three products, which may have implications for their biocompatibility and performance in clinical use.

**Conclusions:**

Gaining a deeper understanding of these differences enables the improved development of dermal fillers to meet the growing demand for safe and effective aesthetic treatments. This study contributes to building knowledge about the use of microparticles in soft tissue fillers for aesthetic treatments and medical applications.

## 1 Introduction

The use of dermal fillers for aesthetic treatments and medical applications has been a topic of great interest and importance in the field of cosmetic dermatology. In recent years, there has been a growing interest in the physical properties of the microparticles used in dermal fillers, particularly on their size, shape, and surface structure, and their impact on biocompatibility and the body's response to the filler. Microparticle morphology significantly impacts tissue integration. Smooth, spherical microparticles promote collagenesis and fibrous tissue development, whereas rough, irregular shapes elicit a foreign body response [1, 2].

Understanding these properties is crucial for the development of new injectable fillers that aim to improve safety and aesthetic outcomes with minimal complications. Calcium hydroxyapatite (CaHA) is a naturally occurring substance in the human body and it is the main mineral substance of teeth and bone. Therefore, it is a biocompatible substance with non‐antigenic properties. Its particles are metabolized by normal homeostatic mechanisms and the specific tests carried out have validated it as a non‐toxic, non‐antigenic, and non‐allergenic implant. The other component of the carrier gel are excipients that have extensive use in medical devices and are classified as “Generally recognized as safe” by the Food and Drug Administration (FDA) [1, 2].

The mechanism of action of CaHA is the formation of collagen through the stimulation of fibroblasts. Histology and immunohistochemistry studies have demonstrated the presence of a local histiocytic and fibroblastic reaction that will lead to the formation of mainly type I and type III collagen [1, 3].

Regarding foreign body reactions, Lemperle et al. evaluated the histological reactions of various fillers injected into the human forearm; no foreign body reaction was reported with the application of calcium hydroxyapatite [4]. This low incidence of granulomas and low potential for foreign body reaction has subsequently been confirmed [5].

CaHA provides immediate and long‐term correction of wrinkles when injected at the subdermal level. The duration of the effect is influenced by various factors, such as the injection site, the application technique, and the patient's metabolism. The average duration of the CaHA effect is 12–18 months [1, 6, 7]. Its use has been first approved for the correction of moderate to severe facial wrinkles and folds and for the treatment of facial lipoatrophy in patients with HIV [6, 7].

The CaHA microspheres do not migrate or calcify; the carrier gel is phagocytosed between 4 and 12 weeks, without calcification [7, 8, 9].

Regarding the induction of biostimulatory effects, CaHA activates collagen, elastin, and angiogenesis from 4 to 9 months following injection, and its duration commonly lasts 9–18 months, while hyaluronic acid (HA) products last 4–6 months. It has been shown that the size, shape regularity, and smoothness of CaHA microspheres of these fillers may vary from each product and affect the patient's physiological and immune response [10]. Recently, a study was carried out using a scanning electron microscope (SEM) to evaluate Radiesse and HArmonyCa particle characteristics, but samples were isolated by washing with miliQ water, then diluted in 10 mL H_2_O and vortexed for 5 min and sedimented by centrifugation. This sample preparation procedure was repeated five times [9]. Regarding particle size, it has been demonstrated that particles with a size < 60 μm in diameter can be engulfed by macrophages and transported to regional lymph nodes [11].

CaHa/CMC dermal filler Radiesse demonstrates both efficacy and safety in its applications, particularly for facial augmentation. It provides immediate aesthetic improvements and contributes to long‐term skin improvement by stimulating collagen production. Patient satisfaction is generally high, indicating that treatment outcomes meet expectations. While side effects are typically infrequent and treatable, nodules are the most commonly reported adverse event, and they often resolve without intervention [9, 10, 11, 12, 13, 14, 15].

HA/CaHa hybrid filler (HArmonyCa) has been shown to be both safe and effective for facial rejuvenation. The filler demonstrated significant improvements in facial volume, tension, and collagen production, leading to positive aesthetic outcomes. Furthermore, HArmonyCa treatment proved to be well‐tolerated, with a low incidence of mostly mild and transient treatment‐emergent adverse events (TEAE). These TEAEs were comparable to those observed with other HA or CaHA dermal fillers and were typical of those expected with this type of procedure. Importantly, only a small fraction of participants (2.5%) experienced TEAEs requiring treatment [16, 17].

No studies on Hydroxyfill were found. The main body of research on calcium hydroxyapatite fillers focuses on Radiesse and, more recently, hybrid fillers like HarmonyCa.

The common adverse events after the injection of CaHA are erythema, edema, and ecchymosis. Tzikas reported the presence of minimal adverse effects (redness, swelling, itching, and bruising); careful patient selection and subepidermal injection significantly reduce these risks [12].

CaHA‐based fillers are widely used for their safety and efficacy; at this time, there is limited data comparing their morphology, which is crucial for understanding their clinical behavior and biocompatibility. This study aims to address this by comparing three products available in México using SEM analysis.

## 2 Materials and Methods

This study analyzed three different dermal filler products, all based on calcium hydroxyapatite microspheres approved for dermal filler procedures in Mexico. The study used SEM analysis to characterize the morphology and characteristics of the calcium hydroxyapatite microspheres used in these dermal filler products.

### 2.1 Materials

The study used three commercially available calcium hydroxyapatite‐based dermal fillers: Hydroxyfill, Radiesse, and HArmonyCa, to isolate and characterize the CaHA microspheres. Despite sharing CaHA as the primary ingredient, the three formulations exhibit some differences.

Radiesse (Merz Pharma) consists of 30% CaHA microspheres (25–45 μm in diameter as reported by the manufacturer) suspended in 70% biodegradable carboxymethyl cellulose (CMC) hydrogel with 0.3% lidocaine, while HArmonyCa (Allergan Aesthetics an AbbVie Company) is a hybrid filler containing 55.7% CaHA microspheres (25–45 μm in diameter as reported by manufacturer), plus 20 mg/mL HA crosslinked with 1,4 butanediol diglycidyl ether (BDDE), also with 0.3% lidocaine. Hydroxyfill (Dr. Korman Laboratories Ltd.) was also analyzed in this study. It is a calcium hydroxyapatite dermal filler consisting of calcium hydroxyapatite microspheres measuring 25–45 μm in diameter, as reported by the manufacturer, (55.7%), glycerin (6.4%), sodium carboxymethyl cellulose (1.3%), and phosphate buffer (36.6%). The percentages are by weight, determined according to the weight obtained.

### 2.2 Scanning Electron Microscope

The calcium hydroxyapatite microsphere samples, obtained from recently manufactured syringes, were air‐dried for 30 min at room temperature to prevent potential fragmentation during subsequent centrifugation. One sample per product was prepared. Following air‐drying, the three samples were gold‐coated under vacuum and analyzed using a Jeol variable pressure Scanning Electron Microscope (model JSM‐6360 LV) at an accelerating voltage of 10 kV to examine particle morphology. For particle size analysis, 10 particle diameters per sample were measured automatically using ImageJ software (version 1.54g). Descriptive statistics and ANOVA were performed using OriginPro software (version 2021 9.8.0.200 Academic) to identify significant differences among the samples.

## 3 Results

The scanning electron microscopic analysis revealed that all three dermal filler products, Radiesse, HArmonyCa, and Hydroxyfill, contained calcium hydroxyapatite microspheres. A one‐way ANOVA was performed to compare the particle size on the product samples, revealing that there was no statistically significant difference in particle sizes between all three samples (*F*(2,27) = 0.329, *p* = [0.722]).

### 3.1 Sample 1

The CaHA/HA (HArmonyCA) microspheres had a consistent and uniform shape and size. They interacted uniformly with their polymeric matrix. When isolated from the HA matrix, they appeared as round, smooth‐surfaced microspheres with an average diameter of 32.54 μm (range of min 27.2 μm, max 38.9 μm).

### 3.2 Sample 2

The scanning electron microscope images of CaHA/CMC (Radiesse) microspheres showed an inhomogeneous surface with a granular texture. Some of the microspheres exhibited small breaks, cracks, and defects, many with irregular and fractured edges. The average size of the CaHA/CMC particles was determined to be 32.51 μm (range of Min 17 μm, Max 43.6 μm) during analysis.

### 3.3 Sample 3

The Hydroxyfill (CaHA/CMC) microspheres had an uneven surface and other residues. SEM images showed that this CaHA/CMC formulation had a uniform distribution of microspheres in the CMC matrix with obvious residues or imperfections. In contrast with samples 1 and 2, SEM images of this CaHA/CMC formulation revealed broken, uneven, and fragmented microspheres with polymer deposits in the cracks. Numerous small microsphere fragments were embedded in the matrix with abundant background residues. In the measurement, the average particle size was 34.68 μm (range of min 25.5 μm, max 46.9 μm).

**FIGURE 1:**
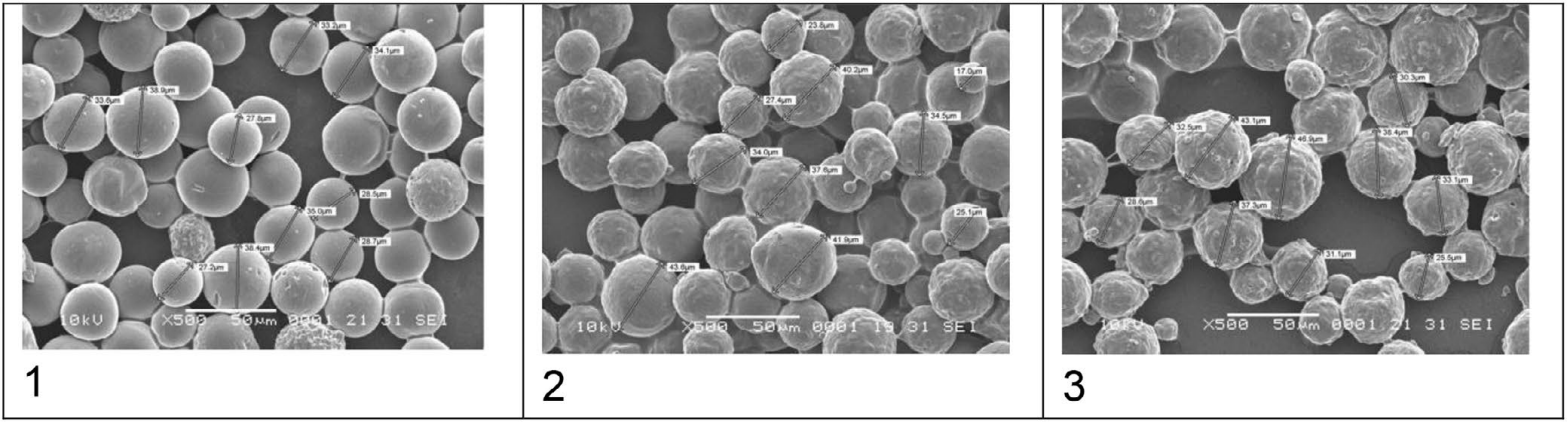
Comparative SEM microphotographs showing HArmonyCA (1), Radiesse (2), and Hydroxyfill (3) ×500.

**FIGURE 2:**
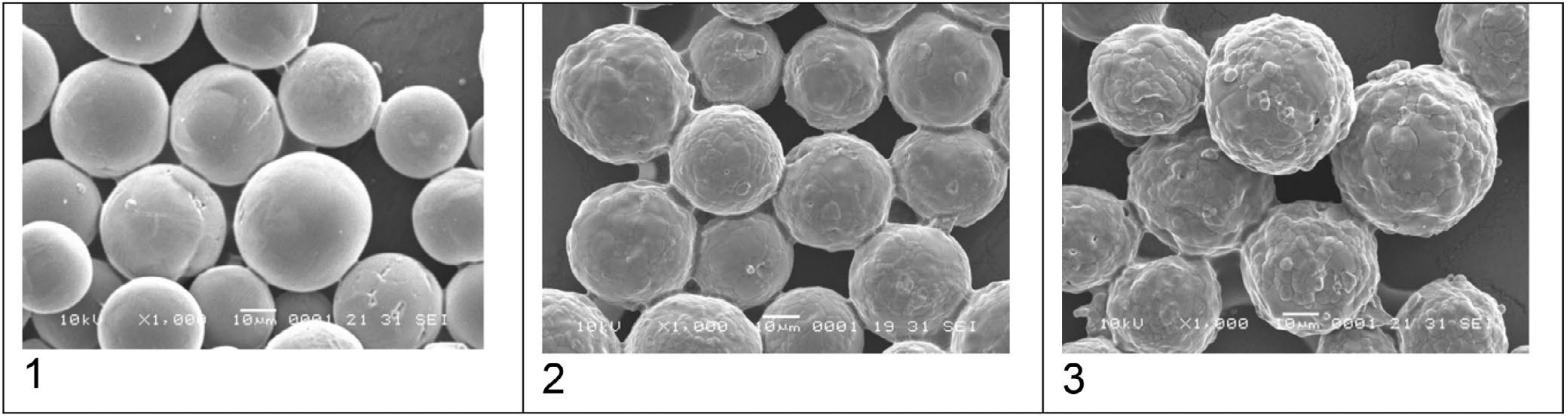
Comparative SEM microphotographs showing surface morphology in HArmonyCA (1), Radiesse (2), and Hydroxyfill (3) ×1000 magnification.

**FIGURE 3:**
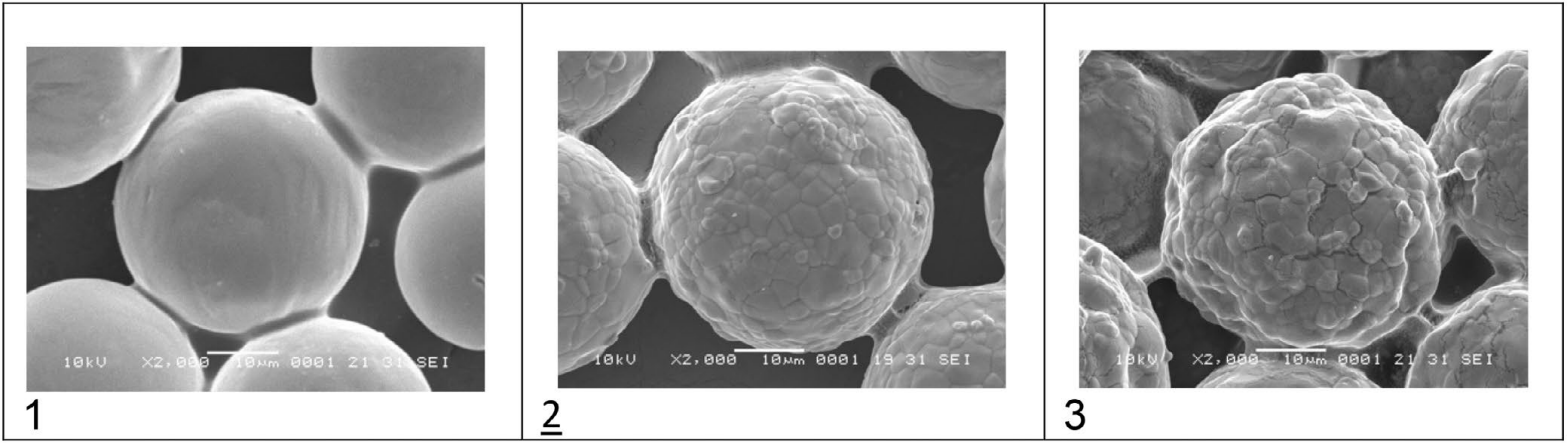
Microphotographs at 2000× comparing HArmonyCA, show an even surface and spherical shape (1) Radiesse shows a slightly rough surface, (2) and, (3) Hydroxyfill shows a rougher surface and a less homogeneous shape.

**FIGURE 4:**
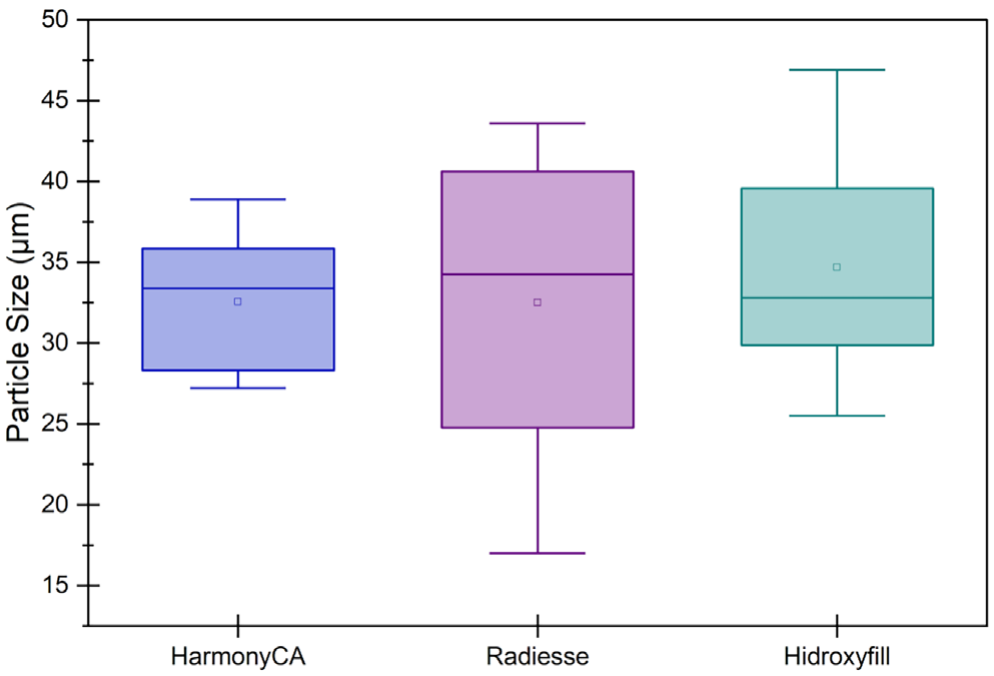
Graphic representation of Min, Max, and average particle size of each product measured in SEM microphotographs comparing HArmonyCA, Radiesse, and Hydroxyfill.

Figure 1 shows that the HArmonyCA sample presents homogeneity between its particles, while the surface of each of them is much more uniform and smooth compared to the other product samples. Figure 2 shows, at 1000×, that the Hydroxyfill particle has a less spherical structure with a lot of roughness on the surface and greater adhesion between particles. Figure 3 shows microphotographs at 2000× comparing the three samples at the larger close‐up. Figure 4 shows the average particle size for the three products, as well as particle size dispersion.

**FIGURE 5:**
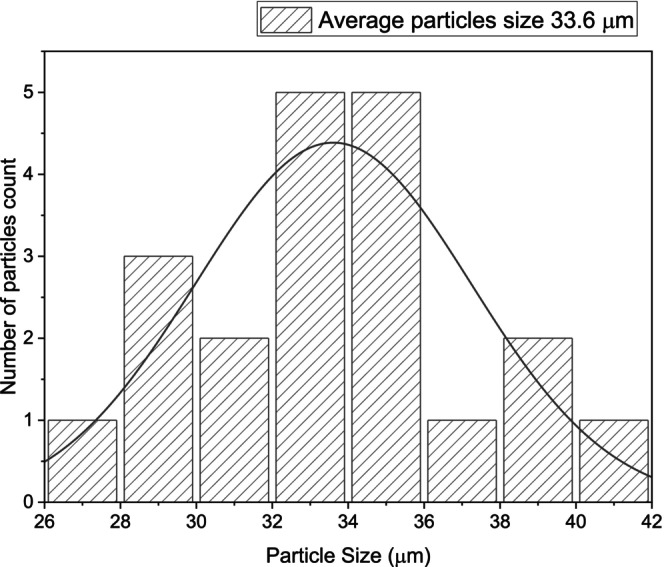
HarmonyCA. Graphic of particle size distribution by number of particles count.

**FIGURE 6:**
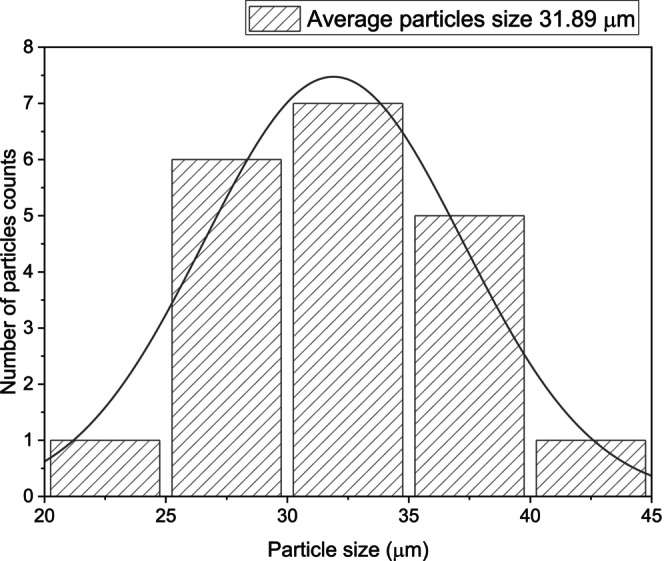
Radiesse. Graphic of particle size distribution by number of particles count.

**FIGURE 7:**
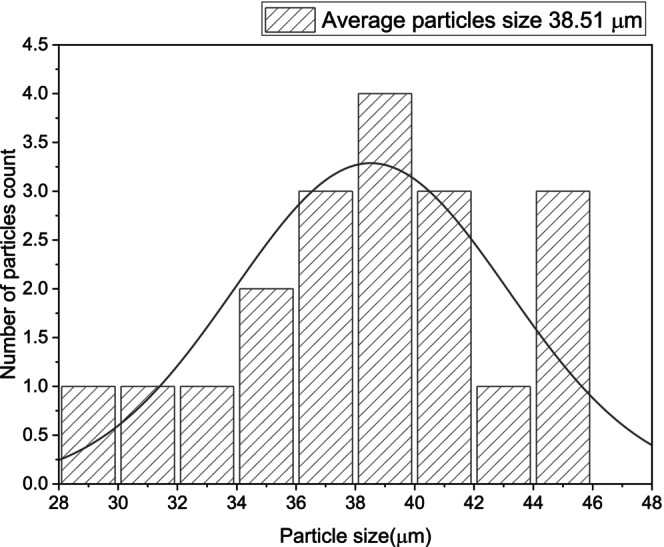
Hidroxyfill. Graphic of particle size distribution by number of particles count.

Figures 5, 6, 7 show the particle size distribution of each product based on the number of particles counted. For HarmonyCA, the mean particle size is 33.60662 μm, with a range from a minimum of 27.486 μm to a maximum of 40.433 μm. For Radiesse, the mean particle size is 31.8964 μm, with a range from a minimum of 23.881 μm to a maximum of 43.438 μm. For Hidroxyfill, the average particle size is 38.51774 μm, with a range from a minimum of 29.06 μm to a maximum of 45.882 μm. Among these, Hidroxyfill exhibits the most uneven particle size distribution and the largest particle size.

## 4 Discussion

The scanning electron microscopic analysis of the three different dermal filler products, Radiesse, HArmonyCA, and Hydroxyfill, revealed different physical properties and sizes of the calcium hydroxyapatite microspheres used in each product. The differences in morphology and characteristics of the microspheres should have important implications for their biocompatibility and performance in clinical use. Direct in vivo studies are needed to clarify this.

The analysis showed that the HArmonyCA microspheres had a consistent and uniform shape and size, appearing as round, smooth‐surfaced microspheres with an average diameter of 32.54 μm. The Hydroxyfill (CaHA/CMC) microspheres showed fragmented particles, uneven surfaces with polymer deposits, broken and fragmented microspheres, and also numerous small microsphere fragments embedded in the matrix and had an average particle size of 34.68 μm with greater variability in size than HArmonyCA. In contrast, the Radiesse microspheres exhibited an inhomogeneous surface with breaks, cracks, and irregular edges, showing greater variability in size and a diameter of 32.51 μm. One limitation of our method was that only one sample per product was analyzed; further studies, including multiple samples from different batches, are needed.

HArmonyCA calcium hydroxyapatite particles are suspended in a crosslinked hyaluronic acid gel and lidocaine, on the other hand, Radiesse and Hydroxyfill use carboxymethylcellulose (CMC) gel as microparticle carriers containing glycerin and a phosphate buffer solution. The variability in the manufacturing processes, the carrier gel selection, and the raw sourcing of calcium hydroxyapatite could explain in part the findings in this study [9, 14].

The fragmentation observed in Hydroxyfill, creating rough surfaces that interact with immune cells, raises concerns about heightened inflammatory responses. This, combined with the inherent variability in microsphere properties among dermal fillers, requires further research to fully understand how these factors influence biocompatibility, overall performance, and ultimately, patient outcomes. Ensuring patient safety and optimal aesthetic results relies on continued clinical investigation into the impact of these morphological variations. Further clinical studies are needed to correlate morphology with clinical outcomes.

## 5 Conclusions

The scanning electron microscopic analysis revealed the morphology and characteristics of the calcium hydroxyapatite microspheres through a sample preparation of air‐drying.

The analysis shows important differences in the physical properties of the microspheres in the three products, which may have implications for their biocompatibility and performance in clinical use. By gaining a deeper understanding of these differences, we can contribute to the development of improved dermal fillers that meet the growing demand for safe and effective aesthetic treatments.

A key factor influencing biocompatibility is the surface structure of these microparticles; smooth‐walled particles are prone to integrate better with surrounding tissues, leading to a milder and more localized inflammatory response. On the other side, microparticles with irregular or rough surfaces can exacerbate inflammation; these uneven surfaces provide a target for the body's immune cells to latch onto, leading to a more pronounced and potentially erratic reaction [13].

Biomaterials that break down naturally over time are less likely to cause chronic inflammation or other complications. This is a key consideration when comparing different filler options, as some are designed to be long‐lasting but not permanently implanted. Clinical studies are needed on the clinical impact of the physical characteristics of these biomaterials.

This study highlights significant differences in the morphology of CaHA microspheres across three dermal fillers, with smoother particles in HArmonyCa potentially offering superior biocompatibility. Future studies are needed to correlate these findings with clinical outcomes to guide product selection and patient safety.

## Conflicts of Interest

The authors declare no conflicts of interest.

## Data Availability

The data that support the findings of this study are available from the corresponding author upon reasonable request.

## References

[1] K. Tansavatdi and D. S. Mangat , “Calcium Hydroxyapatite Fillers,” Facial Plastic Surgery 27, no. 6 (2011): 510–516, 10.1055/s-0031-1298783. 22205523

[2] W. Hubbard , Bioform Implants: Biocompatibility (Bioform, Inc., 2003).

[3] A. L. Berlin , M. Hussain , and D. J. Goldberg , “Calcium Hydroxylapatite Filler for Facial Rejuvenation: A Histologic and Immunohistochemical Analysis,” Dermatologic Surgery 34, no. Suppl 1 (2008): S64–S67. 18547184 10.1111/j.1524-4725.2008.34245.x

[4] G. Lemperle , V. Morhenn , and U. Charrier , “Human Histology and Persistence of Various Injectable Filler Substances for Soft Tissue Augmentation,” Aesthetic Plastic Surgery 27 (2003): 354–366. 14648064 10.1007/s00266-003-3022-1

[5] T. Pavicic , “Calcium Hydroxylapatite Filler: An Overview of Safety and Tolerability,” Journal of Drugs in Dermatology 12, no. 9 (2013): 996–1002. 24002146

[6] L. I. Felderman , “Radiesse for Facial Rejuvenation,” Cosmetic Dermatology 18 (2005): 823–826.

[7] M. Busso and P. L. Karlsberg , “Cheek Augmentation and Rejuvenation Using Injectable Calcium Hydroxylapatite (Radiesse),” Cosmetic Dermatology 19 (2006): 583–588.

[8] R. Mayer , M. Lightfoot , and I. Jung , “Preliminary Evaluation of Calcium Hydroxylapatite as a Transurethral Bulking Agent for Stress Urinary Incontinence,” Urology 57 (2001): 434–438. 11248613 10.1016/s0090-4295(00)01098-0

[9] P. F. Jacovella , “Use of Calcium Hydroxylapatite (Radiesse) for Facial Augmentation,” Clinical Interventions in Aging 3, no. 1 (2008): 161–174. 18488886 10.2147/cia.s2065 PMC2544361

[10] C. Kunzler , C. Hartmann , B. Nowag , et al., “Comparison of Physicochemical Characteristics and Biostimulatory Functions in Two Calcium Hydroxyapatite‐Based Dermal Fillers,” Journal of Drugs in Dermatology 22, no. 9 (2023): 910–916. 37683069 10.36849/JDD.7684

[11] R. A. Ersek and A. A. Beisang, 3rd , “Bioplastique: A New Textured Copolymer Microparticle Promises Permanence in Soft‐Tissue Augmentation,” Plastic and Reconstructive Surgery 87, no. 4 (1991): 693–702. 2008467

[12] T. L. Tzikas , “A 52‐Month Summary of Results Using Calcium Hydroxylapatite for Facial Soft Tissue Augmentation,” Dermatologic Surgery 34, no. Suppl 1 (2008): S9–S15. 18547188 10.1111/j.1524-4725.2008.34237.x

[13] K. Laeschke , “Biocompatibility of Microparticles Into Soft Tissue Fillers,” Seminars in Cutaneous Medicine and Surgery 23, no. 4 (2004): 214–217. 15745227 10.1016/j.sder.2004.09.005

[14] A. Mills and S. Haq , “Harmonyca: A First‐In‐Class, Hybrid, Dual‐Functioning Hyaluronic Acid/Calcium Hydroxyapatite Dermal Filler,” Journal of Aesthetic Nursing 12, no. Sup8 (2023): S6–S12, 10.12968/joan.2023.12.Sup8.S6.

[15] J. A. Kadouch , “Calcium Hydroxylapatite: A Review on Safety and Complications,” Journal of Cosmetic Dermatology 16, no. 2 (2017): 152–161. 28247924 10.1111/jocd.12326

[16] F. Urdiales‐Gálvez , A. Braz , and M. Cavallini , “Facial Rejuvenation With the New Hybrid Filler HArmonyCa™: Clinical and Aesthetic Outcomes Assessed by 2D and 3D Photographs, Ultrasound, and Elastography,” Journal of Cosmetic Dermatology 22, no. 8 (2023): 2186–2197. 37073433 10.1111/jocd.15706

[17] A. Braz , L. Colucci , L. Macedo de Oliveira , et al., “A Retrospective Analysis of Safety in Participants Treated With a Hybrid Hyaluronic Acid and Calcium Hydroxyapatite Filler,” Plastic and Reconstructive Surgery. Global Open 12, no. 2 (2024): e5622. 38348461 10.1097/GOX.0000000000005622 PMC10860969

